# Human XIST: Origin and Divergence of a *cis*-Acting Silencing RNA

**DOI:** 10.3390/ncrna11030035

**Published:** 2025-05-01

**Authors:** Maria Jose Navarro-Cobos, Carolyn J. Brown

**Affiliations:** Department of Medical Genetics, Molecular Epigenetics Group, Life Sciences Institute, University of British Columbia, 2350 Health Sciences Mall, Vancouver, BC V6T 1Z3, Canada; marijo8@student.ubc.ca

**Keywords:** XIST, XCI, lnc-RNAs evolution

## Abstract

Dimorphism of sex chromosomes often leads to a need for dosage compensation. In eutherian mammals, XIST, a long non-coding RNA, is expressed from the X chromosome that will be silenced, triggering X-chromosome inactivation (XCI). XIST originated from the ancestral protein-coding *Lnx3* gene with contributions from various mobile elements that contributed to the striking domains of tandem repeats within the first and sixth exons. Modular domains of XIST are now involved in recruiting heterochromatic marks and proteins essential for XCI initiation and maintenance. This review presents a comparative analysis of human *XIST* with five other eutherian mammals—chimpanzees, cats, pigs, sheep, and mice—examining conservation across exons as well as the tandem repeats. Notably, repeats exhibited higher conservation than exons, underscoring their functional importance. Additionally, a species-specific G repeat, previously described in pigs, was also identified in sheep and cats. These findings provide insights into the domains of XIST, a *cis*-acting silencer that has been used to proposed to alleviate the impact of a supernumerary chromosome in Down syndrome.

## 1. Introduction: Sex Chromosomes, the Need for Dosage Compensation and Long Non-Coding RNAs

In sexually dimorphic organisms, sex can be determined environmentally by the regulation of a sex-determining gene found in both sexes, or it can be genetic, with the sex-determining gene being inherited in one sex [reviewed in [1]]. When genetically determined, the chromosome carrying the sex-determining gene often accumulates further changes, leading to distinguishable sex chromosomes, designated as XY when the female is the homogametic sex (XX) and ZW when the male is the homogametic sex (ZZ). Depending on the extent to which the sex chromosomes diverge, there is a growing requirement for dosage compensation. Sex chromosomes are remarkable in their rapid evolution [1], and thus, many different dosage compensation approaches have emerged. Well-studied examples include hyperactivation of the single X chromosome in the male fruit fly *Drosophila melanogaster*, or the downregulation of both X chromosomes in hermaphrodites *Caenorhabditis elegans* (XX) to equalize expression level to males (X0) [[2] reviewed in [3,4]].

In mammals, Ohno proposed both an upregulation of the X chromosome, and silencing in females [5]. In 1961, Mary Lyon made her prescient hypothesis that one X chromosome in mammalian females was inactivated early in development, and then, this silencing was stable across subsequent cell divisions [6]. The resulting mosaicism of female cells with one or the other X chromosome active is visually apparent in tortoiseshell and calico cats. While subsequent studies have supported Lyon’s hypothesis, the evidence around X-chromosome upregulation remains controversial [reviewed in [7]]. The X inactivation center (*XIC*) is a locus that is required in *cis* for inactivation of that chromosome [8]. During studies to delineate the XIC, the serendipitous discovery of a gene within the *XIC* that is expressed solely from an inactive X (Xi), and thus named *XIST* (Xi-specific transcript), was made [9,10]. Finding of the gene in mice shortly followed [11,12], allowing studies demonstrating that Xist is essential for X inactivation (XCI) [13,14]. XIST was only the second long non-coding RNA (lncRNA) to be identified, after H19 [15]. Elucidation of the full XIST/Xist (human/mouse) transcript revealed striking tandem repeats conserved between the two species [16,17]. With XIST shown to ‘coat’ the Xi [18], the question of how only one of a pair of virtually identical X chromosomes could be silenced within the same nuclear environment shifted to how only a single allele of a gene could be expressed, a question shared with imprinted genes, many of which include lncRNAs [reviewed in [19]]. Additionally, although dosage compensation mechanisms are diverse, lncRNAs reappear as pivotal elements in the process. For example, in *D. melanogaster*, the males upregulate their only X chromosome by the binding of an RNA-protein complex, where the lncRNAs Rox1 and Rox2 bind the MSL proteins on the X chromosome [2] reviewed in [4]. Thus, lncRNAs appear reiteratively in long-range epigenetic gene regulatory processes, raising the question of whether similarities between the structure of lncRNAs contribute to achieving the same function in different species. In this review, we focus on human XIST. Remarkably, XIST appears able to silence in *cis* any chromosome from which it is transcribed [20], and the Lawrence lab has shown that this silencing can be recruited as an agent to silence a supernumerary autosome [21].

Work in our group has attempted to reduce the size of XIST as a functional RNA [22,23], and we have shown that elements from mice can be combined with humans to generate functional silencing molecules [22]. Our results have shown not only the feasibility of generating a shorter fully functional XIST, but also the key importance of some XIST elements; therefore, in this review, we focus on the comparison between human *XIST* and five other mammals to see if key elements such as *XIST* exons or tandem repeats are well-conserved among these species.

## 2. Mammalian X-Chromosome Inactivation

In mammals, the X and Y chromosomes diverged in the therian ancestor ~180 million years ago (MYA) [reviewed in [24]]. The metatherian (marsupial) and eutherian (often called placental) X chromosomes share an X-conserved region (XCR) with the X added region forming much of the short arm of the human X [reviewed in [25]]. Surprisingly, marsupials lack XIST, having instead a lncRNA called Rsx, which is similar to XIST in regulating XCI, being enriched in repeats, and colocalizing with the Xi [26,27]. Interestingly, XIST and Rsx are not conserved, suggesting they evolved independently [reviewed in [28]]. The sex chromosomes are unique amongst the chromosomes, with unique selective pressures. The X chromosome is hemizygous in males, uncovering the impact of new mutations; however, the X chromosome is also present twice as often in females. Relative to other chromosomes, the X chromosome is reported to be enriched in genes involved in functions such as sexual and brain development, and immune system regulation [reviewed in [28,29,30]]. It also has more of the LI retroelement, which has been suggested to participate as a ‘waystation’ for the spread of XCI [31].

XCI initiates early in embryogenesis. In marsupials, XCI is an imprinted process, where the paternal X is always inactivated [reviewed in [32]]. In contrast, in eutherian somatic tissues, XCI so far, has been seen to be random. However, XCI is initially imprinted early in development in mice and rats, and maintained as exclusively paternal inactivation in extra-embryonic tissues. In the mouse epiblast, the Xi reactivates, followed by random XCI [[33], reviewed in [34]]. Other organisms are less extensively studied; however, the process appears to differ considerably across eutheria [35]. In humans, there are fewer studies due to the technical and ethical concerns of accessing pre-implantation embryos; however, there is evidence for early expression from both X chromosomes, which can lead to ‘dampening’ with expression reduced from both X chromosomes [35,36,37].

Human ESCs (hESCs) are present in two states: naïve hESCs, which recapitulate a pre-XCI state with biallelic *XIST* expression, and primed hESCs, which exemplify a post-XCI state, already showing an Xi [37,38]. However, in addition to the mix between naïve and primed cell populations, studies in human ESC have been complicated by a phenomenon called erosion, where the Xi shows reactivation due to epigenetic instability [39,40,41]. Altogether, these findings highlight the limitations of hESCs as reliable models for studying XCI.

*XIST* expression marks the initial step of XCI. This lncRNA is transcribed from the presumptive Xi, coating the chromosome and serving as a scaffold for the recruitment of the essential heterochromatic marks and proteins for XCI establishment and maintenance [reviewed in [42,43,44,45,46]]. Interestingly, the *cis*-regulation of *XIST* expression involves other lncRNAs. In mice, Tsix, an antisense transcript to Xist, acts as a negative regulator. When Tsix expression is disrupted, Xist displays ectopic expression [47,48,49]. In humans, both XIST and TSIX are co-expressed from the Xi, and TSIX does not appear to play a role in XCI [50]. In mice, two lncRNAs, Jpx and Ftx, positively regulate Xist. Jpx is upregulated during XCI and facilitates *Xist* expression by eviction of CTCF from its promoter, which otherwise represses Xist in the pre-XCI state [51,52]. Ftx enhances *Xist* expression by promoting chromatin changes [53]. In humans, both JPX and FTX are present, however, FTX expression is low during pre-implantation, making its participation in XCI improbable. JPX, while expressed at the 2–4 cell stage, distinct from mice, activates XIST in *cis* but appears to act via transcription rather than at the RNA level [54]. Overall, the patterns of early *XIST* expression, regulation, and XCI initiation have changed considerably across eutheria. We now focus on changes within XIST itself.

## 3. XIST Origin: From Ancestral Gene to Mobile Elements

XCI likely emerged early, alongside sex determination by the Y chromosome in mammals, as XIST is conserved among all eutherian mammals. The evolution of *XIST* appears to have been shaped by multiple genetic elements. Interestingly, the XIST gene evolved from a protein-coding gene, *Lnx3*. *Lnx* genes are present across vertebrates and encode ubiquitin E3 ligases. Eutherian mammals possess only two *Lnx1-2* genes since *Lnx* was lost following its pseudogenization, which gave rise to some of the *XIST* exons [55]. In chicken and Xenopus, the *Lnx3* gene is expressed in both females and males in different tissues. There are various reports supporting the hypothesis that some *XIST/Xist* exons evolved from *Lnx3* exons. Duret et al. were the first to report this origin, revealing that human and mouse *XIST* exons 4 and 5 arose from *Lnx3* exons [56]. Elisaphenko et al. concur that *XIST* human and mouse exon 4 and human exon 5 (mouse exon 6) arose from *Lnx3* (exons 4 and 11); and further describe that the *XIST* promoter originated from *Lnx3* exons 1 and 2; mouse exon 5, which is not included in human *XIST*, from *Lnx3* exon 5; and that *Lnx3* exon 3 retains similarity with intron 3 of mice and humans [[57], reviewed in [58]]. While *Lnx3* clearly originated some *XIST/Xist* exons, other genetic elements also contributed to the emergence of the XIST lncRNA gene. These include transposable elements such as endogenous retrovirus and LINEs [57], highlighting the complexity of *XIST* origin.

**Figure 1:**
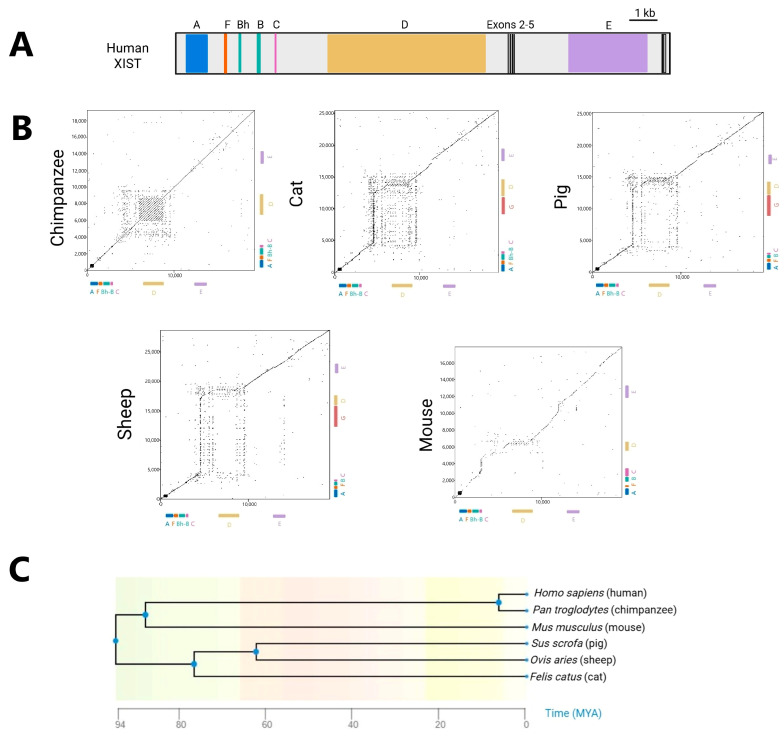
Comparative analysis of XIST conservation across mammals using dot matrix alignments. (**A**) Human XIST transcript short isoform illustrating all the repeats. (**B**) Dot plots comparing human *XIST* transcript against *XIST* predicted transcripts from National Center for Biotechnology Information (https://www.ncbi.nlm.nih.gov/ accessed on 4 April 2025) (Supplementary Table S1) for chimpanzees, pigs, sheep, cats, and mice. Generated using EMBOSS Dotmatcher, parameters with window size = 27 and threshold = 70. (**C**) Timetree (https://timetree.org/ accessed on 10 January 2025) shows the evolutionary distance between 6 eutherian mammals: humans, chimpanzees, mice, pigs, sheep, and cats.

The origin of *XIST* from the addition of retroelements to the ancestral *Lnx3* results in a unique gene structure which in the human structure spans eight exons that can be alternatively spliced and combined with various polyadenylation sites. The processed human transcript is shown in Figure 1A, depicting the shorter of the primary isoforms. The shown variant has splicing from within exon 6 to exons 7 and 8. A short isoform is also observed in mice, and both have been shown to be functional [59,60]. Exons 1 and 6 contain the XIST repeats named A to E that are important for functionality, and are shown in color, with detailed discussion below. The small exons 2 to 5 do not harbor repetitive sequences; however, a recent study reported that exon 5 is important for XCI maintenance [61], while exon 4 has been noted to be amongst the most conserved regions of XIST [62,63], suggesting the possibility that these small exons could be involved in XIST functionality.

## 4. XIST Across Eutheria: Conservation and Divergence

**Figure 2:**
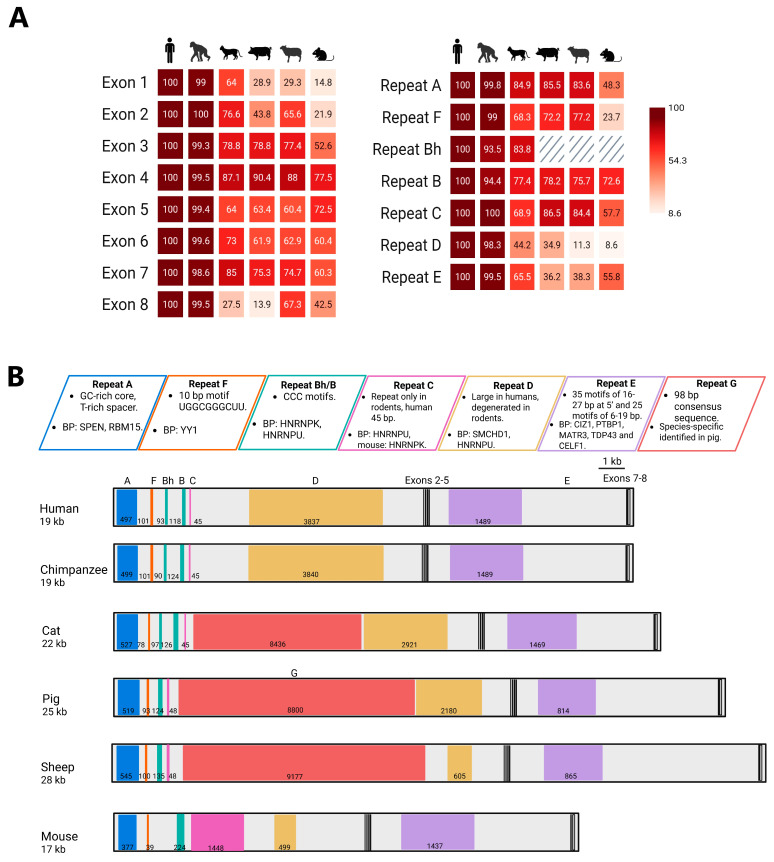
XIST exon and repeat conservation across six eutherian mammals. (**A**) The human conservation scores for exons and repeats. The conservation scores were calculated by multiplying the percentage of sequence identity by the proportion of the human exon or repeat length that aligned, ensuring a normalized comparison across species. For repeat G alignment, the reference was pig repeat G. (**B**) XIST transcripts illustrating the repeats alignments of humans against chimpanzees, cats, pigs, sheep, and mice. BP: binding proteins that interact with these repeats.

In order to compare human XIST to a broader range of eutherians, for Figure 1B, we used EMBOSS Dotmatcher (https://www.ebi.ac.uk/jdispatcher/seqstats/emboss_dotmatcher accessed on 4 April 2025) and *XIST* predicted transcripts from National Center for Biotechnology Information (https://www.ncbi.nlm.nih.gov/ accessed on 4 April 2025). We compared the *XIST* sequences of five evolutionary well-separated mammals—chimpanzees (*Pan troglodytes*), cats (*Felis catus*), pigs (*Sus scrofa*), sheep (*Ovis aries*), and mice (*Mus musculus*)—against the human *XIST* sequence (Supplementary Table S1 lists the accession numbers for *XIST* genes and transcripts). Figure 1C shows the estimated times of divergence of these species. In these dot plots, well-conserved sequences, such as chimps aligned to humans (which is always on the horizontal axis) appear as a diagonal line. Well-conserved repeats, such as the A repeats, appear as a series of diagonal lines, compressed into a box for the short A repeats. Shifts and gaps reflect sequences not conserved, such as the mouse C repeats which are single copy in all other species [64]. Pigs and sheep, which share the same evolutionary node, exhibited similar conservation patterns, with sheep potentially carrying an additional repeat G, as discussed in the next section. The most striking divergence in *XIST* sequences amongst mice, cats, pigs, and sheep, compared to humans, occurred in the region containing repeats C, D, and/or G where substantial gaps and shifts were observed (Figure 1B), highlighting variations in sequence length and content. The mouse *XIST* sequence showed the weakest conservation relative to humans. Overall, there is clear conservation of human XIST with other mammals, with the central repeat regions apparently changing between species. To better visualize the conservation of specific repeats and exons, we generated a comparative human conservation score taking into account both length of match and identity identified by EMBOSS Matcher (Figure 2A, Supplementary Tables S2–S4).

## 5. Conservation of Exons in XIST Evolution

Most studies of *XIST/Xist* have focused on the human or mouse gene. There have, however, been several comparative studies that have identified *XIST* transcripts from other species. Nesterova et al. examined the *Xist* structure across four different vole species (*M. arvalis, M. rossiaemeridionalis, M. kirgisorum*, *and M. transcapicus*) and found strong conservation across the eight exons, with exons 1 and 7 being the largest, with the other five internal exons between 83 and 393 bp in size. Moreover, the vole *Xist* gene is similar to mice and relatively conserved in comparison to human *XIST* [64]. Chureau et al. analyzed *XIST* in humans, mice, and bovine and detected an average conservation between mice versus humans and mice versus bovine of 66% and 62%, respectively. Seven out of eight exons were conserved in the three species, with the further identification of two human-specific exons. Yen sought to identify the coast mole (insectivore) *XIST* but was unable to identify the entire transcript [63]. Hwang et al. described *XIST* in pigs (*Sus scrofa*), which again included seven exons, and notably included an additional distinctive long-repeat called repeat G [65]. Thus, overall retention of exon structures has been observed across examined eutheria.

Using EMBOSS matcher (https://www.ebi.ac.uk/jdispatcher/psa/emboss_matcher accessed on 30 January 2025), each individual XIST exon from humans was aligned against the full predicted genomic *XIST* sequence of chimpanzees, cats, pigs, sheep, and mice (Figure 2A, Supplementary Table S2). Exons 1 and 6 are large, with the internal exons 2 to 5 ranging in length from approximately 60 to 200 bp each. Not surprisingly, chimpanzees showed limited changes from humans, and in general, mice showed the lowest conservation scores with humans. Previous comparisons with humans, mice, and cows have suggested two unique human exons (exon 2 and 7) as well as two mouse exons (2 and 5) which are conserved in other species [66]. These latter exons are therefore not included in our comparison, which is focused on similarity to the human transcript.

Exon 1 spans approximately 11 kb in humans. This exon had the lowest conservation scores among the *XIST* exons largely due to the changes in the repeats, which also impacted exon 1’s size. Exon 2 was the least conserved among the small internal exons, with chimpanzees and cats displaying the highest conservation scores (100 and 76.6, respectively). Sheep had an intermediate level of conservation with a score of 65.5. In contrast, pigs and mice had lower scores of 43.8 and 21.9, respectively. This aligns well with previous reports, in which human exon 2 was found to be absent in cow and rodents, but present in dog [57,63].

For exon 3, the conservation scores were relatively high (99.3, 78.8, 78.8, 77.4, and 52.6, respectively). Exon 4 was the best conserved among all exons, with conservation scores of 99.5, 87.1, 90.4, 88, and 77.5, respectively. This finding is consistent with previous work by Yen et al. [63], and predicting exon 4 could form a stable stem–loop structure, which might explain its high level of conservation [62]. Exon 5 showed only moderate conservation, with scores of 99.4, 64, 63.4, 60.4, and 72.5 across species; however, a somatic deletion of exon 5 was reported to impact maintenance of silencing in a human K562 cell line [61]. Human exon 5 is better conserved with mice exon 5 than in other species. Interestingly, exons 4 and 5 are the ones shown to have been derived from the ancestral Lnx3 gene; however, they show divergent conservation scores. In rabbits, homozygous deletion of exons 2–5 were viable, suggesting these exons are not critical for XCI [67].

Exon 6 conservation scores were derived by using the short isoform exon 6, which is dominated by repeat E (see Figure 1A). Unlike exon 1, where the degree of conservation is lower compared to the repeats it contains, repeat E exhibited similar conservation scores to exon 6. The short isoform splices from just 3′ to repeat E to exon 7, then exon 8. Exon 7 can also be excluded with direct splicing to exon 8. A previous study reported the absence of exon 7 in mice [66]; however, a more recent study that deleted exon 7 in mice ESC observed the delocalization of Xist RNA and an impact on silencing [68]. Exon 7 revealed a moderate conservation with scores of 98.6, 85, 75.3, 74.7, and 60.4, and surprisingly, this exon was the second most conserved exon in cats. Exon 8 had the lowest conservation scores: 99.5, 27.5, 13.9, 67.3, and 42.5, with only sheep showing scores near the level seen for other exons. The lower conservation scores of exons 7 and 8 are consistent with them not being essential for function, since inducible *XIST* transcripts function without them [22,69]. While the majority of the exons are conserved across species, exon 4 is striking in its level of conservation. This conservation was previously noted and led to the Brockdorff group generating a mouse lacking this exon. Surprisingly, there was no detectable effect on XCI, although steady-state levels of Xist were reduced [62]. As a region derived from the original Lnx3 gene, perhaps the exon contributes in a redundant manner to ongoing transcription of the *XIST* gene. This is one of the regions of notable secondary structure, with the other two being tandem repeat regions [70].

## 6. Evolutionary Stability: XIST Repeats Retained Across Species

The tandem repeats are a striking feature of XIST, and using the same approach for exons, the alignment of the XIST tandem repeats using EMBOSS matcher (Figure 2A, Supplementary Tables S3 and S4) demonstrates that the repeats are generally more conserved than the exonic sequences. Protein interaction studies align with approaches studying long-range XIST secondary structures in identifying a modular structure for XIST that overlaps the tandem repeat regions [[71,72] reviewed in [73]].

**Figure 3:**
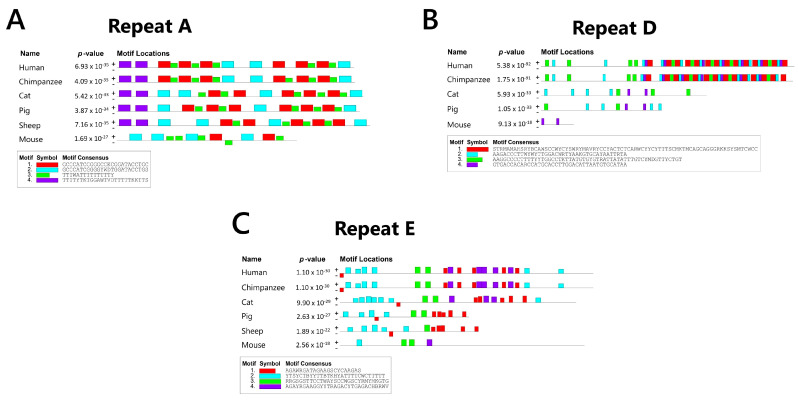
Comparative motif analysis of XIST repeats across six mammals by MEME. (**A**) MEME analysis (https://meme-suite.org/meme/tools/meme accessed on 10 February 2025) of repeat A in humans, chimpanzees, cats, pigs, sheep, and mice. The used parameters were 4 motifs, range: 2–26 nt long. (**B**) MEME analysis of repeat D in humans, chimpanzees, cats, pigs, sheep, and mice. The used parameters were 4 motifs, range: 20–200 nt long. (**C**) MEME analysis of repeat E in humans, chimpanzees, cats, pigs, sheep, and mice. The used parameters were 4 motifs, range: 5–30 nt long.

Repeat A is located near the 5′ end of exon 1 (see Figure 2B) and is comprised of 7.5 to 9 repeats including a well-conserved core and T-rich spacer in all eutherians examined [74]. To investigate sub-motif conservation, we analyzed the repeat A region in our six eutherian mammals using MEME (https://meme-suite.org/meme/tools/meme accessed on 10 February 2025), searching for up to four motifs ranging from 2 to 26 nt in length. The identified motifs were highly consistent in humans, chimpanzees, cats, pigs, and sheep, and simply reflect the conservation of the GC rich core and variability of the T-rich spacer (Figure 3A). Divergence from the human resulted in MEME being unable to detect the 7.5 mouse A repeats. This divergence between humans and mice is also reflected in the conservation scores, where mice had the lowest (48.3), against the rest of the species with conservation scores higher than 80 (Figure 2A).

Deletions of repeat A in mouse embryonic stem cells have shown that it is necessary for gene silencing [75,76,77]. Repeat A is predicted to be highly structured, either by folding into individual stem–loop conformations or by pairing with other A repeats [74,78,79]. One of the key proteins that bind to repeat A in both humans and mice is SPEN, which recruits the NCoR/SMRT complex to activate the histone deacetylase HDAC3 and mediate gene silencing [72,80]. Interestingly, SPEN does not recognize a sequence motif, but inter-repeat helices formed by the repeat A [71]. Another key protein that has been shown to bind repeat A is RBM15, which catalyzes the deposition of the m6A modification that promotes XIST-induced silencing [72,81]. The A repeat region is likely derived from the insertion and duplication of an endogenous retrovirus during XIST evolution from the *Lnx3* gene [57], and it has recently been shown that Spen represses a subset of ERVK elements in mice, suggesting an ancestral function recruited to the Xist RNA [82].

Repeat F is a short 10–40 bp repeat, with only two copies in humans and variable copy numbers across other species [[57,63] reviewed in [73]]. The other species, with the exception of mice, showed strong conservation scores to humans in this region (Figure 2A,B). While little is known about the function of the small repeat, the region surrounding repeat F has multiple YY1 protein binding sites. YY1 is a transcription factor, and the loss of its binding when the repeat F region is deleted in both humans and mice, impacts *XIST* expression [83,84], making it difficult to separate roles for the RNA and the DNA sequence. Another key protein that binds near the repeat F region is LBR, which is anchored to the inner nuclear membrane and facilitates XIST recruitment to the nuclear lamina [85]. This region may be derived from the exaptation of a DNA transposon [57].

Repeat B is a very C-rich region of 6–9 nt repeats, which, in humans, consists of both a 93 bp Bh and a separate 118 bp B repeat region [63] (see Supplementary Table S3 for repeat sequences). Repeat B alignments revealed high human conservation scores among all five species, ranging from 73 to 94. Interestingly, despite cats diverging from humans approximately 90 million years ago (MYA), the Bh repeat—present in both humans and chimpanzees—is also preserved in cat XIST (Figure 1C and Figure 2A,B). Various mouse Xist deletions of repeat B and repeat C demonstrated a significant reduction in two key heterochromatic marks: H3K27me3 and H2AK119ub [77,86,87,88]. The mouse B repeat region has been shown to assemble the Polycomb-initiating complex PCGF3/5-PRC1 via the HNRNPK protein, which subsequently recruits PRC2 [89]. Using transgenes in human somatic cells, we have shown that Bh/B are sufficient to recruit HNRNPK and subsequently, PRC1/2 [23]. The B repeats are 6–9 nt, with a CCC core critical for HNRNPK binding [89]. While repeat Bh is a smaller, separate repeat element that shares sequence similarity with repeat B (but distinct in location and length), they both contribute to humans having 28 CCC motifs in the region. Similar to humans, chimpanzees and cats have 28 and 24 motifs, respectively. In contrast, sheep possess only 14 CCC motifs, however, sheep repeat B is characterized by long uninterrupted stretches of Cs, ranging from 10 to 24 cytosines. This shows that there is variability in the CCC motif density among species.

Repeat C, which may have originated from endogenous retroviruses [57], shows a significant difference in size between humans and mice. Humans repeat C contains less than one motif, while in mice, there are 14 motifs of 115 bp [64]. The alignment of the human C region with chimpanzees, cats, pigs, and sheep revealed high conservation scores and lengths ranging from 45 to 48 bp, supporting the previous evidence of repeat C being rodent-specific [64]. In mice, the targeting of repeat C by locked nucleic acids results in Xist delocalization [90]. In humans, this region, along with repeat B and D, recruits HNRNPU, which is important for localization [72,91]. Other elements may obviate the need for C repeats in other species, and obvious candidates are the D and G repeats, which differ across species.

Repeat D is the largest repeat region in humans, spanning approximately 3.8 kb and consisting of an approximately 200 bp motif repeated eight times, with additional truncated copies. In contrast with repeat C, this repeat is reduced in mice [64]. Human conservation scores were low except in chimpanzees; however, cats and pigs had a similar length to humans. MEME analysis searching for four motifs ranging from 20 to 200 nt supported our conservation scores, finding a strong conservation only with chimpanzee, and only parts of the repeat in other species (Figure 3B). In human somatic cell transgenes, deletions of repeat D impacted the ability of XIST to recruit chromatin modifiers [88]. Consistent with the reduced size and conservation of the mouse D repeats, deletion of the region in mice does not affect Xist cloud formation or PRC enrichment [92]. While such differences might reflect a developmental stage, it also seems possible that repeat D is less needed in mice which has the C repeats. Curiously, the possible origin of repeat D is endogenous retroviruses, similar to repeat C [57].

Repeat E may have evolved from intracisternal A particles [57]. The mouse E repeat is described as containing two subtypes of tandem repeats with the 5′ end harboring 35 C/U/G motifs of 16 to 27 bp, while its 3′ end contains 25 C/U motifs of 6 and 19 bp [reviewed in [73]]. MEME analysis looking for up to four motifs, 5 to 30 bp long, found a similar substructure with a conserved motif at the 5′ end across all species. Additionally, one motif was consistently identified in the central region, while two motifs were present at the 3′ end (Figure 3C). The structure was notably different from mice, although our conservation score was higher for mice than pigs or sheep. Unlike other repeats, repeat E seems to harbor a variety of motifs dispersed along its entire sequence. Repeat E has been reported to be unstructured, rather being a dynamic and flexible platform for binding proteins [93]. Both human and mouse E repeat deletions have shown XIST delocalization. This finding is consistent with the known binding partners of Repeat E, including CIZ1, PTBP1, MATR3, TDP43, and CELF1, all of which are essential for proper XIST/Xist localization [94,95]. Overall, the E repeat region is one of the major hubs of XIST protein binding.

Hwang et al. reported the existence of a unique repeat in pigs, which was named repeat G [65], and it spans approximately between 4 and 13 kb of *XIST* and contains a 98 bp consensus motif. The alignment of the repeat G region exhibited conservation with sheep and cats, with a conservation score of 66 and 61, respectively, suggesting that XIST in both species also bears repeat G. In order to identify the G repeat in other species, we used MAST (https://meme-suite.org/meme/tools/mast accessed on 21 February 2025) with the pig repeat G consensus motif, identifying 43 repeats in pigs, 39 repeats in sheep, and 28 repeats in cats (Supplementary Figure S1). Overall, primates lack G and also C repeats, while cats, sheep, and pigs have this G repeat, but lack the C repeat and are reduced in the size of D. Mice, too, have a reduced D repeat, but also lack G, while having multiple copies of the C repeat region. Thus, it may be that these central repeat units are redundant in their functionality. However, we have been able to synthesize a 5.8 kb XIST that is able to localize and recruit silencing and chromatin modifiers by assembling the A-F, B, and E regions [23], suggesting that in this inducible somatic cell model, C and D (or G) repeats are not required. Recent studies of HNRNPK binding with the B repeat regions highlight that condensates may entrap other proteins recruited by other regions onto the Xi, and such aggregation may enable redundancy across domains [[96] reviewed in [97]].

## 7. Conclusions

In this review, we compared human *XIST* exons and repeat sequences against five other mammalian species. Despite the evolutionary distances between these species, we identified an unexpected conservation of the human Bh repeat in cats and found that the repeat G reported in pigs is also conserved in sheep and cats. However, some repeats or repeat expansions are species-specific (e.g., D-human, C-mouse, G-pig, sheep, and cat) suggesting that functions may be redundant or replaceable. A comparative analysis of exon and repeat conservation revealed that the repeats are more consistently preserved, suggesting that the tandem repeats themselves are more conserved than the surrounding exon sequences. This observation aligns with the functional importance of XIST repeats, which play a critical role in XIST functions and, therefore, must remain conserved to some extent. While research on species other than mice and humans is limited, the conservation of *XIST* across species underscores its significance in XCI.

## Supplementary Materials

The following information can be downloaded at: https://www.mdpi.com/article/10.3390/ncrna11030035/s1, Figure S1: MAST analysis of repeat G. Table S1: XIST validated or predicted genomic and mRNA sequences used for the alignments. Table S2: *XIST* exon alignments. Table S3: Human *XIST* repeats sequences, Table S4: *XIST* repeats alignments.

## Abbreviations

The following abbreviations are used in this manuscript:

ESC	Embryonic stem cells
lncRNAs	Long non-coding RNAs
MYA	Million years ago
XCI	X-chromosome inactivation
XIC	X inactivation center
Xi	Inactive X
