# Supplementary material for: Human XIST: Origin and Divergence of a cis-Acting Silencing RNA
Source: Noncoding RNA. 2025 May 1;11(3):35. doi: 10.3390/ncrna11030035 (PMC12101419; doi:10.3390/ncrna11030035)
Supplement: Supplementary file 1 [file ncrna-11-00035-s001.zip › ncrna-3565435-supplementary.pdf]

Species	Sequence length (bp)	UCSC/NCBI RefSeq
Human	32,103<br>19,296	NG_016172.1<br>NR_001564.2
Chimpanzee	32,082<br>19.247	chrX:69261576-69293657 - (NR_001564)<br>XR_676711.4
Cat	36,329<br>22,425	chrX:62414323-62450651 - (NR_001564)<br>XR_006593313.1
Pig	32,327<br>25,215	KC753464<br>KC753465.1
Sheep	36,211<br>28,706	chrX:62537223-62573433 - (NR_001564)<br>XR_173470.5
Mouse	22,861<br>17,918	NC_000086.8<br>NR_001463.3

Supplementary table 1. XIST validated (human, mouse and pig) or predicted genomic and mRNA sequences used for the alignments.

XIST EXONS ALIGNMENTS	
**EXON 1 (11,173 bp)**	
Human vs chim<br># Length: 11198<br># Homology: 11066/11198 (98.8%)<br># Gaps: 60/11198 (0.5%)	Human vs cat<br># Length: 11989<br># Homology: 7148/11989 (59.6%)<br># Gaps: 2864/11989 (23.9%)
Human vs pig<br># Length: 4554<br># Homology: 3224/4554 (70.8%)<br># Gaps: 530/4554 (11.6%)	Human vs sheep<br># Length: 4652<br># Homology: 3271/4652 (70.3%)<br># Gaps: 480/4652 (10.3%)
Human vs mouse<br># Length: 2638<br># Homology: 1654/2638 (62.7%)<br># Gaps: 281/2638 (10.7%)	
**EXON 2 (64 bp)**	
Human vs chim<br># Length: 64<br># Homology: 64/64 (100.0%)<br># Gaps: 0/64 (0.0%)	Human vs cat<br># Length: 64<br># Homology: 49/64 (76.6%)<br># Gaps: 7/64 (10.9%)
Human vs pig<br># Length: 42<br># Homology: 28/42 (66.7%)<br># Gaps: 2/42 (4.8%)	Human vs sheep<br># Length: 65<br># Homology: 42/65 (64.6%)<br># Gaps: 5/65 (7.7%)
Human vs mouse<br># Length: 22<br># Homology: 14/22 (63.6%)<br># Gaps: 0/22 (0.0%)	
**EXON 3 (137 bp)**	
Human vs chim<br># Length: 137	Human vs cat<br># Length: 136

# Homology: 136/137 (99.3%)<br># Gaps: 0/137 (0.0%)	# Homology: 108/136 (79.4%)<br># Gaps: 0/136 (0.0%)
Human vs pig<br># Length: 137<br># Homology: 108/137 (78.8%)<br># Gaps: 1/137 (0.7%)	Human vs sheep<br># Length: 137<br># Homology: 106/137 (77.4%)<br># Gaps: 0/137 (0.0%)
Human vs mouse<br># Length: 125<br># Homology: 72/125 (57.6%)<br># Gaps: 12/125 (9.6%)	
**EXON 4 (209 bp)**	
Human vs chim<br># Length: 209<br># Homology: 208/209 (99.5%)<br># Gaps: 0/209 (0.0%)	Human vs cat<br># Length: 207<br># Homology: 182/207 (87.9%)<br># Gaps: 7/207 (3.4%)
Human vs pig<br># Length: 206<br># Homology: 189/206 (91.7%)<br># Gaps: 1/206 (0.5%)	Human vs sheep<br># Length: 209<br># Homology: 184/209 (88.0%)<br># Gaps: 1/209 (0.5%)
Human vs mouse<br># Length: 203<br># Homology: 162/203 (79.8%)<br># Gaps: 3/203 (1.5%)	
**EXON 5 (164 bp)**	
Human vs chim<br># Length: 164<br># Homology: 163/164 (99.4%)<br># Gaps: 0/164 (0.0%)	Human vs cat<br># Length: 169<br># Homology: 105/169 (62.1%)<br># Gaps: 44/169 (26.0%)
Human vs pig<br># Length: 170<br># Homology: 104/170 (61.2%)<br># Gaps: 43/170 (25.3%)	Human vs sheep<br># Length: 154<br># Homology: 99/154 (64.3%)<br># Gaps: 38/154 (24.7%)
Human vs mouse<br># Length: 169<br># Homology: 119/169 (70.4%)<br># Gaps: 10/169 (5.9%)	
**EXON 6 (1,872 bp)**	
Human vs chim<br># Length: 1872<br># Homology: 1865/1872 (99.6%)<br># Gaps: 0/1872 (0.0%)	Human vs cat<br># Length: 2031<br># Homology: 1367/2031 (67.3%)<br># Gaps: 317/2031 (15.6%)
Human vs pig<br># Length: 1923<br># Homology: 1159/1923 (60.3%)<br># Gaps: 475/1923 (24.7%)	Human vs sheep<br># Length: 1934<br># Homology: 1178/1934 (60.9%)<br># Gaps: 436/1934 (22.5%)
Human vs mouse	

# Length: 1907<br># Homology: 1131/1907 (59.3%)<br># Gaps: 352/1907 (18.5%)<br># Score: 1687	
**EXON 7 (146 bp)**	
Human vs chim<br># Length: 146<br># Homology: 144/146 (98.6%)<br># Gaps: 0/146 (0.0%)	Human vs cat<br># Length: 147<br># Homology: 124/147 (84.4%)<br># Gaps: 6/147 (4.1%)
Human vs pig<br># Length: 146<br># Homology: 110/146 (75.3%)<br># Gaps: 2/146 (1.4%)	Human vs sheep<br># Length: 145<br># Homology: 109/145 (75.2%)<br># Gaps: 5/145 (3.4%)
Human vs mouse<br># Length: 132<br># Homology: 88/132 (66.7%)<br># Gaps: 7/132 (5.3%)	
**EXON 8 (374 bp)**	
Human vs chim<br># Length: 374<br># Homology: 372/374 (99.5%)<br># Gaps: 0/374 (0.0%)	Human vs cat<br># Length: 177<br># Homology: 103/177 (58.2%)<br># Gaps: 18/177 (10.2%)
Human vs pig<br># Length: 59<br># Homology: 52/59 (88.1%)<br># Gaps: 5/59 (8.5%)	Human vs sheep<br># Length: 345<br># Homology: 252/345 (73.0%)<br># Gaps: 29/345 (8.4%)
Human vs mouse<br># Length: 263<br># Homology: 159/263 (60.5%)<br># Gaps: 54/263 (20.5%)<br># Score: 259	

Supplementary table 2. XIST exon alignments human vs chimpanzee, cat, pig, sheep and mouse using EMBOSS Matcher. "Homology" represents the values given as identity and similarity by EMBOSS Matcher.

**HUMAN XIST REPEATS SEQUENCES**
REPEAT A<br>GGAATTTTTCTTTGGAATCATTTTTGGTTGACATCTCTGTTTTTTGTGGATCAGTTTTTTACTCTTCCACTCTCTTTT<br>CTATATTTTGCCCATCGGGGCTGCGGATACCTGGTTTTATTATTTTTTCTTTGCCCAACGGGGCCGTGGATACCT<br>GCCTTTTAATTCTTTTTTATTCGCCCATCGGGGCCGCGGATACCTGCTTTTTATTTTTTTTTCCTTAGCCCATCGGG<br>GTATCGGATACCTGCTGATTCCCTTCCCCTCTGAACCCCCAACACTCTGGCCCATCGGGGTGACGGATATCTGCT<br>TTTTAAAAATTTTCTTTTTTTGGCCCATCGGGGCTTCGGATACCTGCTTTTTTTTTTTTTATTTTTCCTTGCCCATCG<br>GGGCCTCGGATACCTGCTTTAATTTTTGTTTTTCTGGCCCATCGGGGCCGCGGATACCTGCTTTGATTTTTTTTTT<br>TCATCGCCCATCGGTGCTTTTTATGGATGAAAAAATGT

REPEAT F<br>TGTTGGGTTTTGCCGCAGGGACAATATGGCAGGCGTTGTCATATGTATATCATGGCTTTTGTCACGTG<br>GACATCATGGCGGGCTTGCCGCATTGTTAAAGA
Repeat Bh<br>ATACCTCCCCCCCCACCCCCCAACCCCCCCAACTCCCCACCCCCACCCCCCACCCCCCACCTCCCCACCC<br>CCCTACCCCCCTACCCCCCTACC
Repeat B<br>CCTCCCCAGCCCTGCTCCCAGCAAACCCCTAGTCTAGCCCCAGCCCTACTCCCACCCCGCCCCAGCCCT<br>GCCCCAGCCCCAGTCCCCTAACCCCCCAGCCCTAGCCCCAGTCCCAGTC
Repeat C<br>GGCATTGCTGATCTTCAGTACTGACTCCTTGACCATTTTCAGTTA
Repeat D<br>TTCCATTAAATTAAGATCCCAACTGCTCACACCCTCTTAGCATTACAGTAGAGGGTGCTAATCACAAGGACATTT<br>CTTTTGTACTGTTAATGTGCTACTTGCATTTGTCCCTCTTCCTGTGCACTAAAGACCCCACTCACTTCCCTAGTGTT<br>CAGCAGTGGATGACCTCTAGTCAAGACCTTTGCACTAGGATAGTTAATGTGAACCATGGCAACTGATCACAACA<br>ATGTCTTTCAGATCAGATCCATTTTATCCTCCTTGTTTTACAGCAAGGGATATTAATTACCTATGTTACCTTTCCCT<br>GGGACTATGAATGTGCAAAATTCCAATGTTCATGGTCTCTCCCTTTAAACCTATATTCTACCCCTTTTACATTATA<br>GAAAGGGATGCTGGAAACCCAGAGTCCTTCTCTTGGGACTCTTAATGTGTATTTCTAATTATCCATGACTCTTAA<br>TGTGCATATTTTCAATTGCCTAATTGATTTCAATTGTCTAAGACATTTCAAATGTCTAATTGATTAGAACTGAGTC<br>TTTTATATCAAGCTAATATCTAGCTTTTATATCAAGCTAATATCTTGACTTCTCAGCATCATAGAAGGGGGTACTG<br>ATTTCCTAAAGTCTTTCTTGAATTTCTATTATGCAAAATTGCCCTGAGGCCGGGTGTGGTGGCTCACACCTGTAA<br>TCCCAGCACTTTGGGAGGCTGAGGTGGGAAGATCCCTTACTGCCAGGAGTTTGAGACCAGCCTGGCCAACATT<br>AAAAAAAAAAAAAAAGTAAGACAATTGCCCTGGAATCCCATCCCCCTCACACCTCCTTGGCAAAGCAGCAGGAGT<br>GCTAACTAGCTAGTGCTTCTTCTCTTATACTGCTTAAATGCGCATAATTAGCAGTAGTTGATGTGCCCCTATGTTA<br>GAGTAGAATCCCGCTTCCTTGCTCCATTTGCATTACTGCAGGAGCTTCTAACTAGCCTGAATTCACTCTCTTGGAC<br>TGTTAATGTGCATACTTATATTTGCTGCTGTACTTTTTTACCATGTAAGGACCCCACCCACTGTATTTACATCCCA<br>GCTGGAAGTACCTACTACTTAAGACCCTTAGACTAGTAAAGTTAGCGTGCATAATCTTAGGTGTTATATACACAT<br>TTTCAGTTGCATACAGTTGTGCCTTTTATCAGGACTCCTGTACTTATCAAAGCAGAGAGTGCTAATCAATATTAA<br>GCCCTTCTCTTCGAACTGTAGATGGCATGTAATTGCAGTTGTCAATGGTCCTTCAATTAGACTTGGGTTTCTGAC<br>CTATCACACCCTCTTTGCTTTATTGCATGGGGTACTATTCACTTAAGGCCCCTTTCTCAAACTGTTAATGTGCCTA<br>ATGACAATTACATCAGTATCCTTCCTTTTGAAGGACAGCATGGTTGGTGACACCTAAGGCCCCATTTCTTGGCCT<br>CCCAATATGTGTGATTGTATTTGTCGAGGTTGCTATGCACTAGAGAAGGAAAGTGCTCCCCTCATCCCCACTTTT<br>CCCTTCCAGCAGGAAGTGCCCACCCCATAAGACCCTTTTATTTGGAGAGTCTAGGTGCACAATTGTAAGTGACC<br>ACAAGCATGCATCTTGGACATTTATGTGCGTAATCGCACACTGCTCATTCCATGTGAATAAGGTCCTACTCTCCG<br>ACCCCTTTTGCAATACAGAAGGGTTGCTGATAACGCAGTCCCCTTTTCTTGGCATGTTGTGTGTGATTATAATCG<br>TCTGGGATCCTATGCACTAGAAAAGGAGGGTCCTCTCCACATACCTCAGTCTCACCTTTCCCTTCCAGCAGGGAG<br>TGCCCACTCCATAAGACTCTCACATTTGGACAGTCAAGGTGCGTAATTGTTAAGTGAACACAACCATGCACCTTA<br>GACATGGATTTGCATAACTACACACAGCTCAACCTATCTGAATAAAATCCTACTCTCAGACCCCTTTTGCAGTAC<br>AGCAGGGGTGCTGATCACCAAGGCCCTTTTTCCTGGCCTGGTATGCGTGTGATTATGTTTGTCCCGGTTCCTGTG<br>TATTAGACATGGAAGCCTCCCCTGCCACACTCCACCCCCAATCTTCCTTTCCCTTCCGGCAGGGAGTGCCCTCTCC<br>ATAAGACGCTTACGTTTGGACAATCAAGGTGCACAGTTGTAAGTGACCACAGGCATACACCTTGGACATTAATG<br>TGCATAACCACTTTGCCCATTCCATCTGAATAAGGTCCTACTCTCAGACCCCTTTTGCAGTACAGCAGGGGTGCT<br>GATCACCAAGGCCCCTTTTCTTGGCCTGTTATGTGCGTGATTATATTTGTCTGGGTTCCTGTGTATTAGACAAGG<br>AAGCCTTCCCCCCGCCCCCACCCCCACTCCCAGTCTTCCTTTCCCTTCCAGCAGGGAGTGCCCCCTCCATAAGATC<br>ATTACATTTGGACAATCAAGGTGCACAATTATAAGTGACCACAGCCATGCACCTTGGACATTATTGGACATTAAT<br>GTGCGTAACTGCACATGGCCCATCCCATCTGAATAAGGTCCTACTCTCAGATGCCCTTTGCAGTACAGCAGGGG<br>TACTGAATCACCAAGGCCCTTTTTCTTGGCCTGTTATGTGTGTGATTATATTTATCCCAGTTTCTGTGTAATAGAC

ATGAAAGCCTCCCCTGCCACACCCCACCTCCAATCTTCCTTTCCCTTCCACCAGGGAGTGTCCACTCCATATACCC<br>TTACATTTGGACAATCAAGGTGCACAATTGTAAGTGAGCATAGGCACTCACCTTGGACATGAATGTGCATAACT<br>GCACATGGCCCATCCCATCTGAATAAGGTCCTACTCTCAGACCCTTTTTGCAGTACAGCAGGGGTGCTGATCACC<br>AAGGCCCCTTTTCCTGGCCTGTTATGTGTGTGATTATATTTGTTCCAGTTCCTGTGTAATAGACATGGAAGCCTC<br>CCCTGCCACACTCCACCCCCAATCTTCCTTTCCCTTCTGGCAGGAAGTACCCGCTCCATAAGACCCTTACATTTGG<br>ACAGTCAAGGTGCACAATTGTATGTGACCACAACCATGCACCTTGGACATAAATGTGTGTAACTGCACATGGCC<br>CATCCCATCTGAATAAGGTCCTACTCTCAGACCCCTTTTGCAGTACAGTAGGTGTGCTGATAACCAAGGCCCCTC<br>TTCCTGGCCTGTTAACGTATGTGATTATATTTGTCTGGGTTCCAGTGTATAAGACATGGAAGCCTCCCCTGCCCC<br>ACCCCACCCTCAATCTTCCTTTCCCTTCTGGCAGGGAGTGCCAGCTCCATAAGAACCTTACATTTGGACAGTCAA<br>GGTGCACAATTCTAAGTGACCGCAGCCATGCACCTTGGTCAATAATGTGTGTAACTGCACACGGCCTATCTCAT<br>CTGAATAAGGCCTTACTCTCAGACCCCTTTTGCAGTACAGCAGGGGTGCTGATAACCAAGGCCCATTTTCCTGG<br>CCTGTTATGTGTGTGATTATATTTGTCCAGGTTTCTGTGTACTAGACAAGGAAGCCTCCTCTGCCCCATCCCATCT<br>ACGCATAATCTTTCTTTTCCTCCCAGCAGGGAGTGCTCACTCCATAAGACCCTTACATTTGGACAATCAAGGTGC<br>ACAATTGTAAGTGACCACAACCATGCATCTTGGAAATTTATGTGCATAACTGCACATGGCTTATCCTATTTGAAT<br>AAAGTCCTACTCTCAGACCCCCTTTGCAGTATAGCTGGGGTGCTGATCACTGAGGCCTCTTTGCTTGGCTTGTCT<br>ATATTCTTGTGTACTAGATAAGGGCACCTTCTCATGGACTCCCTTTGCTTTTCAACAAGGAGTACCCACTACTTTT<br>TAAGA
Repeat E<br>CTTTTTGTCCTTTTCTTGTTCTGTCTACCTCTCCTTTCTCTGCCTACCTCTCTTTTCTCTTTGTGAACTGTGATTATTT<br>GTTACCCCTTCCCCTTCTCGTTCGTTTTAAATTTCACCTTTTTTCTGAGTCTGGCCTCCTTTCTGCTGTTTCTACTTT<br>TTATCTCACATTTCTCATTTCTGCATTTCCTTTCTGCCTCTCTTGGGCTATTCTCTCTCTCCTCCCCTGCGTGCCTCA<br>GCATCTCTTGCTGTTTGTGATTTTCTATTTCAGTATTAATCTCTGTTGGCTTGTATTTGTTCTCTGCTTCTTCCCTTT<br>CTACTCACCTTTGAGTATTTCAGCCTCTTCATGAATCTATCTCCCTCTCTTTGATTTCATGTAATCTCTCCTTAAATA<br>TTTCTTTGCATATGTGGGCAAGTGTACGTGTGTGTGTGTCATGTGTGGCAGAGGGGCTTCCTAACCCCTGCCTG<br>ATAGGTGCAGAACGTCGGCTATCAGAGCAAGCATTGTGGAGCGGTTCCTTATGCCAGGCTGCCATGTGAGATG<br>ATCCAAGACCAAAACAAGGCCCTAGACTGCAGTAAAACCCAGAACTCAAGTAGGGCAGAAGGTGGAAGGCTC<br>ATATGGATAGAAGGCCCAAAGTATAAGACAGATGGTTTGAGACTTGAGACCCGAGGACTAAGATGGAAAGCC<br>CATGTTCCAAGATAGATAGAAGCCTCAGGCCTGAAACCAACAAAAGCCTCAAGAGCCAAGAAAACAGAGGGT<br>GGCCTGAATTGGACCGAAGGCCTGAGTTGGATGGAAGTCTCAAGGCTTGAGTTAGAAGTCTTAAGACCTGGGA<br>CAGGACACATGGAAGGCCTAAGAACTGAGACTTGTGACACAAGGCCAACGACCTAAGATTAGCCCAGGGTTGT<br>AGCTGGAAGACCTACAACCCAAGGATGGAAGGCCCCTGTCACAAAGCCTACCTAGATGGATAGAGGACCCAAG<br>CGAAAAAGGTATCTCAAGACTAACGGCCGGAATCTGGAGGCCCATGACCCAGAACCCAGGAAGGATAGAAGC<br>TTGAAGACCTGGGGAAATCCCAAGATGAGAACCCTAAACCCTACCTCTTTTCTATTGTTTACACTTCTTACTCTTA<br>GATATTTCCAGTTCTCCTGTTTATCTTTAAGCCTGATTCTTTTGAGATGTACTTTTTGATGTTGCCGGTTACCTTTA<br>GATTGACAGTATTATGCCTGGGCCAGTCTTGAGCCAGCTTTAAATCACAGCTTTTACCTATTTGTTAGGCTATAG<br>TGTTTTGTAAACTTCTGTTTCTATTCACATCTTCTCCACTTGAGAGAGACACCAAAATCCAGTCAGTATCTAATCT<br>GGCTTTTGTTAACTTCCCTCAGGAGCAGACATTCATATAGGTGATACTGTATTTCAGTCCTTTCTTTTGACCCCAG<br>AAGCCCTAGACTGAGAAGATAAAATGGTCAGGTTGTTGGGGAAAAAAAAGTGCCAGGCTCTCTA

Supplementary table 3. Human XIST repeats sequences used for the alignments against chimpanzee, cat, pig, sheep and mouse.

**XIST REPEATS ALIGNMENTS**	
**REPEAT A**	
**CHIMPANZEE (284-782)**<br># Length: 499<br># Homology: 496/499 (99.4%)	**CAT (277-793)**<br># Length: 527<br># Homology: 422/527 (80.1%)

# Gaps: 2/499 (0.4%)	# Gaps: 45/527 (8.5%)
**PIG (220-728)**<br># Length: 519<br># Homology: 425/519 (81.9%)<br># Gaps: 34/519 (6.6%)	**SHEEP (272-802)**<br># Length: 545<br># Homology: 416/545 (76.3%)<br># Gaps: 62/545 (11.4%)
**MOUSE**<br># Length: 366<br># Homology: 240/366 (65.6%)<br># Gaps: 51/366 (13.9%)	
**REPEAT F**	
**CHIMPANZEE (1444-1544)**<br># Length: 101<br># Homology: 100/101 (99.0%)<br># Gaps: 0/101 (0.0%)	**CAT (1445-1522)**<br># Length: 78<br># Homology: 69/78 (88.5%)<br># Gaps: 1/78 (1.3%)
**PIG (1389-1481)**<br># Length: 93<br># Homology: 73/93 (78.5%)<br># Gaps: 1/93 (1.1%)	**SHEEP (1449-1548)**<br># Length: 100<br># Homology: 78/100 (78.0%)<br># Gaps: 1/100 (1.0%)
**MOUSE**<br># Length: 39<br># Homology: 24/39 (61.5%)<br># Gaps: 0/39 (0.0%)	
**REPEAT Bh**	**REPEAT B**
**CHIMPANZEE (1976-2064)**<br># Length: 90<br># Homology: 87/90 (96.7%)<br># Gaps: 1/90 (1.1%)	**CHIMPANZEE (2808-2931)**<br># Length: 124<br># Homology: 117/124 (94.4%)<br># Gaps: 6/124 (4.8%)
**CAT (2031-2126)**<br># Length: 97<br># Homology: 78/97 (80.4%)<br># Gaps: 8/97 (8.2%)	**CAT (2894-3018)**<br># Length: 126<br># Homology: 96/126 (76.2%)<br># Gaps: 9/126 (7.1%)
	**PIG (2664-2785)**<br># Length: 124<br># Homology 97/124 (78.2%)<br># Gaps: 8/124 (6.5%)
	**SHEEP (2745-2878)**<br># Length: 135<br># Homology: 94/135 (69.6%)<br># Gaps: 18/135 (13.3%)
	**MOUSE**<br># Length: 118<br># Homology: 90/118 (76.3%)<br># Gaps: 9/118 (7.6%)
**REPEAT C**	
**CHIMPANZEE (3050-3094)**<br># Length: 45<br># Homology: 45/45 (100.0%)	**CAT (3227-3271)**<br># Length: 45<br># Homology: 31/45 (68.9%)

# Gaps: 0/45 (0.0%)	# Gaps: 4/45 (8.9%)
**PIG (2954-3001)**<br># Length: 48<br># Homology: 39/48 (81.2%)<br># Gaps: 4/48 (8.3%)	**SHEEP (2995-3042)**<br># Length: 48<br># Homology: 38/48 (79.2%)<br># Gaps: 4/48 (8.3%)
**MOUSE**<br># Length: 38<br># Homology: 26/38 (68.4%)<br># Gaps: 5/38 (13.2%)	
**REPEAT D**	
**CHIMPANZEE (4587-8402)**<br># Length: 3840<br># Homology: 3782/3840 (98.5%)<br># Gaps: 27/3840 (0.7%)	**CAT (12452-14958)**<br># Length: 2921<br># Homology: 1697/2921 (58.1%)<br># Gaps: 583/2921 (20.0%)
**PIG (13085-14916)**<br># Length: 2180<br># Homology: 1341/2180 (61.5%)<br># Gaps: 383/2180 (17.6%)	**SHEEP (17210-17791)**<br># Length: 605<br># Homology: 434/605 (71.7%)<br># Gaps: 29/605 (4.8%)
**MOUSE**<br># Length: 205<br># Homology: 136/205 (66.3%)<br># Gaps: 16/205 (7.8%)	
**REPEAT E**	
**CHIMPANZEE (25035-26523)**<br># Length: 1489<br># Homology: 1482/1489 (99.5%)<br># Gaps: 0/1489 (0.0%)	**CAT (29639-31025)**<br># Length: 1469<br># Homology: 975/1469 (66.4%)<br># Gaps: 227/1469 (15.5%)
**PIG (25964-26721)**<br># Length: 814<br># Homology: 539/814 (66.2%)<br># Gaps: 72/814 (8.8%)	**SHEEP (29321-310135)**<br># Length: 865<br># Homology: 572/865 (66.1%)<br># Gaps: 93/865 (10.8%)
**MOUSE**<br># Length: 1437<br># Homology: 832/1437 (57.9%)<br># Gaps: 263/1437 (18.3%)	
**PIG REPEAT G**	
**4-13Kb mRNA Pig vs mRNA Human**<br># Length: 281<br># Homology: 177/281 (63.0%)<br># Gaps: 22/281 (7.8%)	**4-13Kb mRNA Pig vs mRNA Chim**<br># Length: 183<br># Homology: 121/183 (66.1%)<br># Gaps: 2/183 (1.1%)
**4-13Kb mRNA Pig vs mRNA Cat**<br>(4634-12614)<br># Length: 8436<br># Homology: 5288/8436 (62.7%)<br># Gaps: 643/8436 (7.6%)	**4-13Kb mRNA Pig vs mRNA Sheep**<br>(8566-17106)<br># Length: 9110<br># Homology: 5636/9110 (61.9%)<br># Gaps: 952/9110 (10.5%)
**4-13Kb mRNA Pig vs mRNA mouse**<br># Length: 64	

# Homology: 46/64 (71.9%)<br># Gaps: 3/64 (4.7%)	

Supplementary Table 4. XIST repeats alignments human vs chimpanzee, cat, pig, sheep and mouse, and pig repeat G vs human, chimpanzee, cat, sheep and mouse using EMBOSS matcher. “Homology” represents the values given as identity and similarity by EMBOSS Matcher.

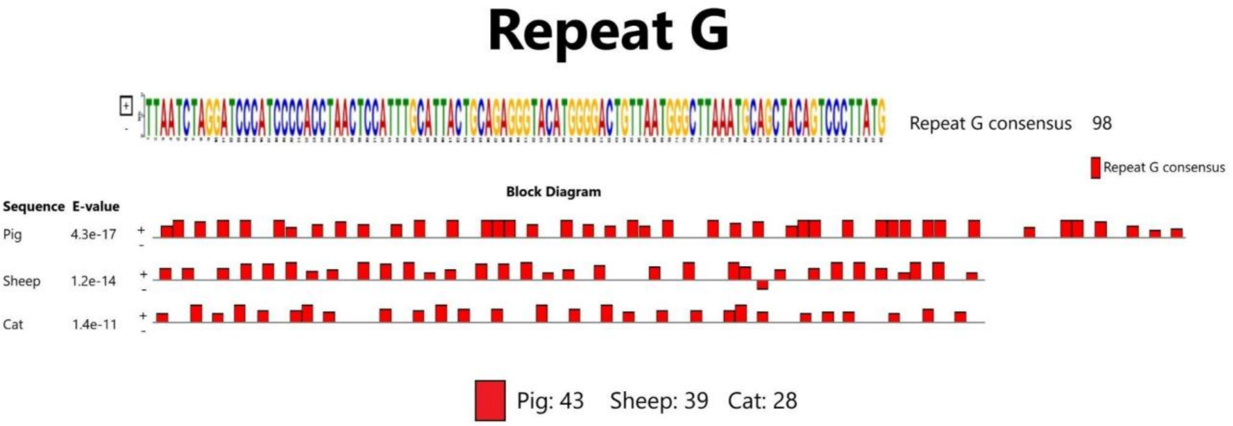

Supplementary Figure 1. MAST analysis (https://meme-suite.org/meme/tools/mast) of pig repeat G, the consensus sequence of repeat G was searched in pig, sheep and cat.
